# Histone gene expression and histone mRNA 3' end structure in *Caenorhabditis elegans*

**DOI:** 10.1186/1471-2199-8-51

**Published:** 2007-06-14

**Authors:** Rebecca Keall, Sandra Whitelaw, Jonathan Pettitt, Berndt Müller

**Affiliations:** 1 Institute of Medical Sciences, School of Medical Sciences, University of Aberdeen, Foresterhill, Aberdeen AB25 2ZD, Scotland, UK

## Abstract

**Background:**

Histone protein synthesis is essential for cell proliferation and required for the packaging of DNA into chromatin. In animals, histone proteins are provided by the expression of multicopy replication-dependent histone genes. Histone mRNAs that are processed by a histone-specific mechanism to end after a highly conserved RNA hairpin element, and lack a poly(A) tail. In vertebrates and *Drosophila*, their expression is dependent on HBP/SLBP that binds to the RNA hairpin element. We showed previously that these *cis *and *trans *acting regulators of histone gene expression are conserved in *C. elegans*. Here we report the results of an investigation of the histone mRNA 3' end structure and of histone gene expression during *C. elegans *development.

**Results:**

Sequence analysis of replication-dependent histone genes revealed the presence of several highly conserved sequence elements in the 3' untranslated region of histone pre-mRNAs, including an RNA hairpin element and a polyadenylation signal. To determine whether in *C. elegans *histone mRNA 3' end formation occurs at this polyadenylation signal and results in polyadenylated histone mRNA, we investigated the mRNA 3' end structure of histone mRNA. Using poly(A) selection, RNAse protection and sequencing of histone mRNA ends, we determined that a majority of *C. elegans *histone mRNAs lack a poly(A) tail and end three to six nucleotides after the hairpin structure, after an A or a U, and have a 3' OH group. RNAi knock down of CDL-1, the *C. elegans *HBP/SLBP, does not significantly affect histone mRNA levels but severely depletes histone protein levels. Histone gene expression varies during development and is reduced in L3 animals compared to L1 animals and adults. In adults, histone gene expression is restricted to the germ line, where cell division occurs.

**Conclusion:**

Our findings indicate that the expression of *C. elegans *histone genes is subject to control mechanisms similar to the ones in other animals: the structure of *C. elegans *histone mRNA 3' ends is compatible with histone-specific mRNA 3' end processing; CDL-1 functions in post-transcriptional control of histone gene expression; and *C. elegans *histone mRNA levels are elevated at periods of active cell division, indicating that histone gene expression is linked to DNA replication.

## Background

Packaging of DNA into macromolecular nucleoprotein complexes is essential for cell division and the control of gene expression. In eukaryotes, histone proteins associate with DNA to form chromatin. Chromatin formation occurs during S phase and is dependent on the coordination of histone and DNA synthesis. In animals, replication-dependent histone genes are expressed during S phase and provide the protein building blocks for chromatin. They are the only genes known to produce mRNAs that lack polyadenylated (poly (A)) tails, and instead end with a highly conserved hairpin structure. Specialised histones, termed replacement variant histones, define specialised chromatin domains and are independently integrated into chromatin. The expression of replacement variant histone genes is not linked to DNA synthesis, and their mRNAs, which normally lack the hairpin element, are polyadenylated.

In animals, the four core histone proteins H2A, H2B, H3 and H4 and the linker histone H1 are normally encoded by multi copy replication-dependent histone genes (hereafter simply referred to as histone genes). These genes are clustered and have conserved histone specific elements in the 3' untranslated region (3'UTR), including the hairpin RNA element. The RNA binding protein termed hairpin-binding, or stem-loop binding protein (HBP/SLBP) specifically binds the RNA hairpin and is a key regulator of histone gene expression [1,2]. HBP/SLBP facilitates histone mRNA 3' end processing in the nucleus [2-4], and controls histone mRNA translation [5-7]. An additional motif, the histone downstream element or spacer element (HDE), is present 3' of the hairpin and serves as binding site for the U7 small nuclear ribonucleoprotein (U7 snRNP). U7 snRNP binding occurs via base pairing of the U7 snRNA, the RNA component of the U7 snRNP, to the HDE and is facilitated by HBP/SLBP. It is instrumental for the formation of the histone mRNA 3' end by endonucleolytic cleavage after the hairpin, most likely by U7snRNP- mediated recruitment of the RNA endonuclease CPSF-73 [8,9]. As in mammals, *Drosophila *dSLBP is essential and is required for histone-specific mRNA 3' end processing [10,11]. In dSLBP mutants, histone-specific mRNA 3' end processing is inhibited and end formation occurs instead via cryptic polyadenylation signals and results in polyadenylated histone mRNA [11,12]. However, this alternative RNA 3' end processing is not sufficient to complement the defect in histone gene expression in dSLBP mutants.

**Figure 1:**
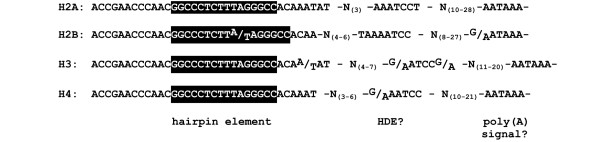
**Conserved sequence elements in the 3'UTR of *C. elegans *histone genes**. 3' UTR regions of histone H2A, H2B, H3 and H4 genes were downloaded from Wormbase, aligned using ClustalW and the conserved sequence elements were identified (the alignment of histone gene 3'UTRs is shown in additional file 1). The consensus sequences for the 3'UTR elements of histones H2A, H2B, H3 and H4 are shown separately. The hairpin element, the core of the binding site for CDL-1, is boxed in black. A conserved element with the core sequence AATCC located at the position of a putative U7 snRNP binding site (HDE?), and a polyadenylation signal sequence are indicated. The 32–36 nucleotide region encompassing the hairpin structure is absolutely conserved at nearly all positions. The only significant deviation from the consensus is found in the hairpin loop of four histone H2B genes where the sequence is CTTA instead of CTTT. The core AATCC element is absolutely conserved in most histone genes except for H3 genes, where the first nucleotide is a G instead of an A in 5 out of 13 sequences. Other deviations from the consensus are minor and normally involve only one nucleotide. The core sequence is embedded in longer conserved elements that differ between the different histone gene types. The AATAAA polyadenylation signal is absolutely conserved in H2A genes, 11 out of 14 H2B genes, 12 out of 13 H3 genes and 11 out of 14 H4 genes. The other H2B, H3 and H4 genes have previously described variants of this sequence [39]. Further elements involved in polyadenylation are a CA dinucleotide at the cleavage site and a G/GU rich downstream element. Such elements can be found downstream of the polyadenylation signal, but their position and, in case of the G/GU rich element, their sequence are less well conserved (see also additional file 1).

*C. elegans *has multicopy H2A, H2B, H3 and H4 histone genes that have hallmarks of replication-dependent histone genes: they are clustered and have a conserved hairpin structure in the 3'UTR [13]. This element is followed by a highly conserved potential U7snRNP binding site [14], and then immediately by a conserved polyadenylation signal (Figure 1 and additional file 1). All known *C. elegans *histone H1 genes are similar to replacement variant histone genes and do not have the conserved 3'UTR sequence elements [15]. We previously identified the sole *C. elegans *HBP/SLBP CDL-1 [2,13], and showed that it binds specifically to the *C. elegans *hairpin RNA element [16]. *cdl-1 *is essential and required for histone synthesis [13,17].

Here we investigate the expression of *C. elegans *histone genes during development and elucidate the structure of *C. elegans *histone mRNA 3' ends. We observed that, as is the case in other metazoans, histone mRNAs end after the hairpin structure and not after the conserved polyadenylation signal, compatible with histone mRNA 3' end formation by histone-specific processing. We demonstrate that knockdown of *cdl-1 *severely inhibits histone gene expression, but does not have a significant effect on histone mRNA steady state levels, compatible with a role for *cdl-1 *in post-transcriptional control of histone mRNA. Finally we report that histone gene expression in *C. elegans *correlates with the presence of actively dividing cells, consistent with a need to generate histones during cell division.

## Results

### The structure of *C. elegans *histone mRNAs 3' end structure

A survey of *C. elegans *histone gene 3'UTRs and flanking regions revealed that the conserved 16 nucleotide hairpin sequence, the core element of the binding site for CDL-1 [16], is embedded in a highly conserved 32–36 nucleotide region, and is followed by a similarly conserved AATCC element at a position indicating that this element may act as HDE, the binding site for the U7 snRNP (Fig. 1 and additional file 1). In addition, all histone genes have at least one polyadenylation signal AATAAA motif, or a variant thereof, immediately downstream of the AATCC. This is followed by a CA dinucleotide that may act as cleavage site, and G/T or T-rich downstream sequences that may act as a downstream element in polyadenylation (additional file 1) [18]. Thus, clearly identifiable and potential histone-specific 3'UTR sequence elements (hairpin element and possible HDE, respectively) and polyadenylation signals are conserved and in close proximity to each other. Polyadenylation signals are also found in other animals downstream of histone genes. For example, a survey of the human H2A histone genes revealed that canonical polyadenylation signals are located at distances from between 102 to > 400 nucleotides downstream of the histone hairpin. In *Drosophila*, histone genes are organised in tandem repeats with intergenic regions that contain polyadenylation signals at various distances from the hairpin, and these signals are used for mRNA 3' end processing in a dSLBP mutant background [12]. In the presence of dSLBP, *Drosophila *histone mRNAs end shortly after the hairpin, similar to vertebrate histone mRNA [19-21].

**Figure 2:**
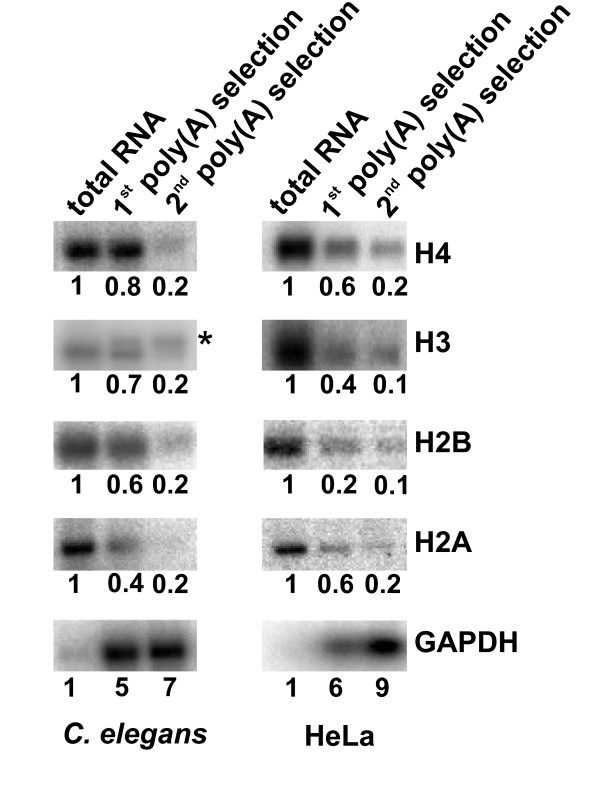
**The structure of histone mRNA 3' ends: poly(A) selection of histone mRNA**. Northern analysis of poly(A) selected RNA. Total RNA isolated from adult N2 animals and from Hela cells was subjected to two successive rounds of poly(A) selection using oligo-dT cellulose. 2 μg of total RNA and of poly(A) selected RNA were analysed by Northern blotting and histone H2A, H2B, H3, H4, and GAPDH mRNA levels were determined as described. Blots were stripped between probings. RNA levels were measured as described and were expressed as fold enrichment, with the RNA levels in total mRNA defined as 1. The asterisk marks the position of a H3 mRNA species enriched during poly(A) selection of *C. elegans *RNA.

We wished to determine whether histone mRNA 3' end processing would result in mRNAs ending after the hairpin as in other metazoans, or whether 3' end formation would normally occur by cleavage and polyadenylation using the conserved polyadenylation signal, and result in polyadenylated mRNA. First, we exploited the fact that polyadenylated histone mRNA is enriched by selection with oligo-dT coupled to cellulose [22,23]. Total RNA prepared from adult N2 animals was isolated and subjected to two subsequent rounds of selection with oligo-dT cellulose, and the RNA content was analysed by Northern blotting (Fig. 2). For comparison we performed the same procedure in parallel with total RNA extracted from HeLa cells. Polyadenylated human and *C. elegan*s GAPDH mRNA were enriched, indicating that the selection procedures were successful. As expected, human histone mRNAs, which are known to lack a poly(A) tail [24,25] and do not to bind to oligo(dT) cellulose [26,27], were lost during the selection for poly(A) RNA. *C. elegans *histone mRNAs were similarly lost during the procedure, indicating that the structure of *C. elegans *histone mRNA 3' ends is similar to human histone mRNA ends. An exception was the enrichment of a low mobility *C. elegans *histone H3 mRNA species that may represent polyadenylated transcripts of *C. elegans *replacement variant histone H3 genes [13].

**Figure 3:**
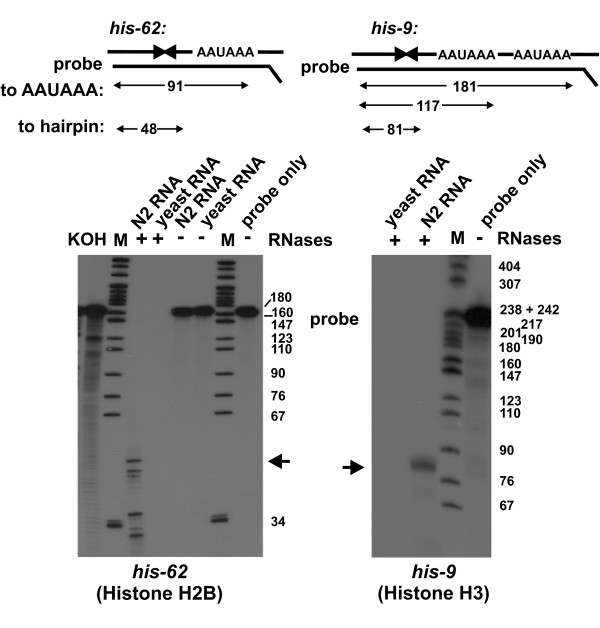
**The structure of histone mRNA 3' ends: RNase protection assays**. Top: schematic representation of RNase protection assays showing protected fragments produced when histone mRNAs end after the hairpin structure or after the AAUAAA polyadenylation signals. *his-62*: internally ^32^P labelled *his-62 *probe was hybridised with either total RNA from N2 animals or yeast RNA and subjected to treatment with RNase A/T1 as described, where indicated. Probe marks radioabelled *his-62 *probe. Size standards were ^32^P labelled pBR322 *Msp*I marker (M) and a KOH single nucleotide ladder (KOH) produced from 5' ^32^P end labelled probe. Note that this combination of RNA and DNA marker does not allow for a precise determination of fragment length. We based our estimate on DNA marker fragments and used the nucleotide ladder to determine the fragment length between the 34 and 67 nucleotide DNA marker fragments. The ~50 nucleotide product resulting from hybridisation to mRNA ending after the hairpin is indicated by the arrow. Analysis was by denaturing PAGE followed by autoradiography as described. *his-9*: the internally^32^P labelled *his-9 *probe was hybridised with total RNA from N2 animals or yeast RNA, and subjected to treatment with RNAse A/T1. Size standard (M) was ^32^P labelled pBR322 *Msp*I DNA. Probe marks radioabelled *his-9 *probe. The major ~80 nucleotide product is indicated by the arrow. Analysis was as for *his-62 *RNase protection assay.

Next, we performed RNase protection assays to further characterise the ends of selected histone mRNA species (Fig. 3). Probes were designed to distinguish between mRNAs from the H2B gene *his-62 *and the histone H3 genes *his-9, his-13 and his-42 *ending after the polyadenylation signal or after the hairpin structure, respectively. The 3'UTR and 3' flanking regions of the *his-62 *H2B gene contains the highly conserved 32 nucleotide element encompassing the hairpin element, and AATCC and AATAAA elements, but differs in the non-conserved regions from other H2B genes. RNase protection with the *his-62 *probe revealed a series of protected fragments. Using a combination of DNA marker and single-nucleotide ladder produced by KOH treatment of 5' end labelled probe RNA to estimate the length of the protected fragments, we found that the longest protected fragment was ~50 nucleotides long. This is compatible with protection of the probe by hybridisation with *his-62 *mRNA ending after the hairpin. Shorter protected fragments were most likely due to partial protection of the probe by hybridisation to other histone transcripts. Fragments of ~32 to ~36 nucleotides length can be accounted for by hybridisation to the region containing the hairpin element conserved in the other H2B mRNAs and also in mRNAs from other histone gene types (see Fig. 1), and longer fragments of ~46 and ~47 nucleotides most likely arose from hybridisation of the probe to other H2B mRNAs that differ from *his-62 *mRNA at the 5'end of the protected region (between the termination codon and the conserved element containing the hairpin element).

The 3'UTR of the *his-9 *gene is identical to the 3'UTRs of the *his-13 *and *his-42 *genes, which are therefore also detected in RNase protection assays with the probe designed to determine the *his-9 *mRNA 3' end. RNase protection with total RNA prepared from N2 adults and the *his-9 *probe revealed protected histone mRNA fragments of ~81 nucleotides in length, indicating that the majority of transcripts from the *his-9*, *his-13 *and *his-42 *genes lacked the polyadenylation signal and ended after the hairpin. Thus, the findings obtained with two experimental approaches described – poly(A) selection and RNase protection – are compatible with the majority of *C. elegans *histone mRNAs ending after the hairpin structure and lacking a poly(A) tail.

To independently confirm this we decided to determine the sequence of *C. elegans *histone mRNA 3' ends. We exploited the fact that yeast poly(A) polymerase will add non-template encoded nucleotides to the 3' end of RNA molecules with a 3'OH group [28]. We first incubated total RNA prepared from adult N2 animals with inosine triphosphate (ITP) and yeast poly(A) polymerase, and subsequently added ATP to the reaction. RNAs were then reverse transcribed using an oligo-dT/anchor primer and reverse transcriptase. Histone 3'UTR fragments were PCR amplified using histone-specific primers and an anchor primer, cloned and sequenced (Table 1). All fragments sequenced were derived from histone mRNAs, demonstrating that our protocol was specific and that the histone mRNAs had a 3'OH group. Comparison with genomic DNA sequence revealed that all fragments contained the conserved hairpin structure. Significantly, they diverged from genomic DNA three to six nucleotides downstream of the hairpin structure, after an A or after a T, where a non-genome encoded tail was observed. In mRNA 3' end formation by polyadenylation, the poly(A) tail is added 3' of the AAUAAA signal. As this element was not present in any of the sequences analysed, this was in agreement with the results described in Figure 3 and confirms that histone mRNAs 3' ends were not formed by a canonical cleavage/polyadenylation reaction. While in many cases the tail consisted of As only, a significant proportion of these tails started with Gs, the result of the addition of inosine, marking the precise position of the mRNA end. As incorporation of inosine by poly(A) polymerase is very inefficient compared to the incorporation of adenosine, the proportion of sequences where the tails start with inosine is an underrepresentation of the proportion of mRNAs ending after the hairpin [28]. In conclusion, this approach showed that histone mRNAs end three to six nucleotides 3' of the hairpin structure, after an A or after a U, and have a 3'OH group. This is similar to vertebrate, sea urchin and *Drosophila *histone mRNAs that normally end a few nucleotides after the hairpin, 3' of an A and also have a 3'OH group [19,20,29]. We cannot completely exclude the possibility that some *C. elegans *histone mRNA ends – represented by sequences where inosine was inserted into a poly(A) tail – have poly(A) tails added after the hairpin structure *in vivo *that were then extended during the experiment by incorporation of first inosine and then adenosine. Polyadenylated histone mRNAs with a short poly(A) tail added immediately after the hairpin have been detected in *Xenopus *oocytes [30]. The tail is lost after fertilisation, concomitant with the translational activation of histone mRNA [31]. However, the combination of poly(A) selection, RNase protection assays and sequence analysis indicates that the majority of *C. elegans *histone mRNA do not extend past the polyadenylation signal but end three to six nucleotides after the hairpin structure. This is compatible with histone mRNA 3' ends being formed by histone mRNA cleavage after the hairpin, as in other animals [19-21,29].

**Table 1:** Sequence determination of histone mRNA 3' ends.

**Histone Type**	**No. clones**	**Genomic Sequence**	**Non-genome encoded tail**
H3	1	AAC*GGCCCTCTTTAGGGCC*ACAT	G_[8]_AA_ [75]_
	1	AAC*GGCCCTCTTTAGGGCC*ACAT	G_[2]_AA_ [162]_
	1	AAC*GGCCCTCTTTAGGGCC*ACA	GAA_ [102]_
	1	AAC*GGCCCTCTTTAGGGCC*ACATAT	AG_[9]_AA_ [107]_
	3	AAC*GGCCCTCTTTAGGGCC*ACA	AA_ [67–96]_
	1	AAC*GGCCCTCTTTAGGGCC*ACTT	A_[9]_GAA_ [92]_
	1	AAC*GGCCCTCTTTAGGGCC*ACA	A_[31]_GAA_ [119]_
	1	AAC*GGCCCTCTTTAGGGCC*ACA	A_ [53]_GAA_ [84]_
			
H2A	1	AAC*GGCCCTCTTTAGGGCC*ACAT	G_[4]_AGAA_ [88]_
	1	AAC*GGCCCTCTTTAGGGCC*ACAAA	GAA_ [96]_
	7	AAC*GGCCCTCTTTAGGGCC*ACA	AA_ [62–95]_
	1	AAC*GGCCCTCTTTAGGGCC*ACAT	AA_ [88]_
	1	AAC*GGCCCTCTTTAGGGCC*ACATT	AA_ [47]_
	1	AAC*GGCCCTCTT-AGGGCC*ACA	A_[14]_GAA_ [84]_
	1	AAC*GGCCCTCTTTAGGGCC*ACA	A_[22]_GAA_ [45]_
	1	AAC*GGCCCTCTTTAGGGCC*ACA	A_ [83]_GAA_[31]_
	1	AAC*GGCCCTCTTTAGGGCC*ACA	A_ [49]_GGAA_[39]_
			
H2B	1	AAC*GGCCCTCTTTAGGGCC*ACAA	GAA_ [42]_
	2	AAC*GGCCCTCTTTAGGGCC*ACATT	GGAA_ [55,63]_
	12	AAC*GGCCCTCTTTAGGGCC*ACA	AA_ [61–135]_
	1	AAC*GGCCCTCTTTAGGGCC*ACAT	AA_ [86]_
	1	AAC*GGCCCTCTTTAGGGCC*ACT	AA_ [77]_
	1	AAC*GGCCCTCTTTAGGGCC*ACA	A_ [54]_GAA_[34]_
	1	AAC*GGCCCTCTTTAGGGCC*ACA	A_ [51]_GAA_[24]_
			
H4	1	AAC*GGCCCTCTTTAGGGCC*ACTT	GAA_ [114]_
	10	AAC*GGCCCTCTTTAGGGCC*ACA	AA_ [37–108]_
	3	AAC*GGCCCTCTTTAGGGCC*ACAT	AA_ [54–115]_
	1	AAC*GGCCCTCTTTAGGGCC*ACATT	AA_ [90]_
	2	AAC*GGCCCTCTTTAGGGCC*ACTT	AA_ [20,74]_

Histone mRNA was tailed with ITP and subsequently ATP using poly(A) polymerase, and histone mRNA 3'UTRs were amplified by RT-PCR, cloned and sequenced. Sequences were aligned using the hairpin structure as anchor (in italic). The hairpin structure is followed by genome encoded nucleotides and a non-genome encoded tail added by poly(A) polymerase. The transition point from genomic sequence to non-genomic tail marks the 3' end of histone mRNA that is located three to six nucleotides after the hairpin.

### Knockdown of CDL-1 inhibits histone protein synthesis but does not affect histone mRNA levels

**Figure 4:**
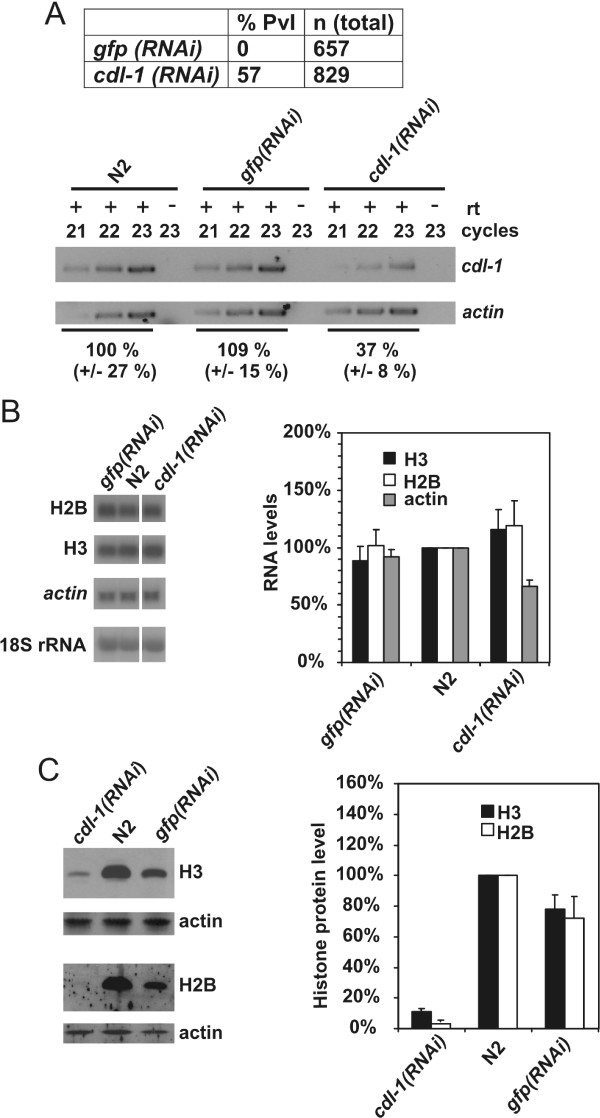
***cdl-1 *mRNA knockdown by RNAi prevents histone synthesis but does not affect steady-state histone mRNA levels**. N2 animals were either *cdl-1 (RNAi) *or control *gfp(RNAi) *treated and grown to adults as described, when either RNA was prepared or animals were lysed for protein analysis. Similarly, N2 animals were grown to adults and subjected to RNA isolation or lysis for protein analysis. (A) top. Summary of phenotypes observed. Pvl refers to protruding vulva. bottom. *cdl-1 *mRNA knockdown was confirmed by comparison of RNA levels in *cdl-1(RNAi) *animals to RNA levels untreated (N2) and in *gfp(RNAi) *N2 animals, by reverse transcription followed by 21–23 cycles of PCR. Separate amplification of actin mRNA was used as control. Control reactions where reverse transcription was omitted (- rt) were performed in parallel. RNA levels were quantitated as described in Methods. (B) Actin mRNA, Histone H3 and H2B mRNA, and 18S rRNA levels in untreated N2 animals, *cdl-1(RNAi) *or *gfp(RNAi) *animals were analysed by Northern blotting. RNAs were analysed on different part of the same blot. The blot was stripped between probings for histone mRNAs. mRNA levels were standardised with respect to 18S rRNA levels and compared to levels of RNA in N2 animals, which was defined as 100 %. The graph shows mean data from 2 independent experiments, with the error bar indicating the maximum value. (C) Levels of actin, histone H3 and histone H2B proteins in *cdl-1(RNAi)*, *gfp(RNAi) *and N2 animals were determined by Western blotting. For the graph, H3 and H2B protein levels were standardised with respect to actin levels, and compared to levels in N2 animals which were defined as 100 %. The average and maximum value of two independent measurements are shown.

We previously described the identification of the *C. elegans *HBP/SLBP CDL-1 and reported that it is essential for histone gene expression and cell division [2,13,16]. We observed that *cdl-1(RNAi) *and RNAi targeting histone H3 and H4 genes resulted both in sterility and early embryonic arrest, linked to defects on chromatin structure [2]. We wished to extend this study by determining whether the reduction of histone proteins in *cdl-1(RNAi) *treated animals was linked to a reduction in steady state histone mRNA levels. Animals were subjected to *cdl-1(RNAi) *by feeding. Untreated animals and *gfp(RNAi) *animals were used as controls. As reported previously, depletion of *cdl-1 *expression using the same RNAi-by-feeding regime resulted in a protruding vulva (Pvl) phenotype [13]. This phenotype was observed in 57 % of *cdl-1(RNAi) *animals, but not in *gfp(RNAi) *animals which were wild type in appearance (Fig. 4A). This was paralleled by an ~60 % reduction of *cdl-1 *mRNA in *cdl-1(RNAi) *animals compared to N2 and *gfp(RNAi) *animals, as determined by semi-quantitative RT-PCR (Fig. 4A).

We then examined the effect of *cdl-1(RNAi) *and *gfp(RNAi) *on histone H2B and H3 gene expression. We expect, given the similarities of histone genes and examples from other species, that findings are transferable to the other replication-dependent histone genes. Histone H2B and H3 mRNA levels were not significantly different in animals subjected to the two RNAi treatments, and were similar to levels in untreated animals (Fig. 4B). For comparison we also determined actin mRNA levels and could again not find a significant, more then 2-fold change in mRNA level. To determine whether the *cdl-1(RNAi) *treatment caused RNA 3' end formation to occur more frequently at polyadenylation signals, we compared the structure of *his-62 *mRNA in RNA prepared from N2 animals and from *cdl-1(RNAi) *animals (additional file 2). This assay did not provide evidence for a change in histone mRNA 3' end formation in *cdl-1(RNAi) *animals. Analysis of histone protein levels by Western blotting revealed that histone H2B and H3 levels were broadly similar in untreated and *gfp(RNAi) *N2 animals (Fig. 4C). Relative histone protein levels plotted in the graph in Figure 4C were standardised using actin as reference. We have currently no explanation for the ~30 % difference in relative histone protein levels between *gfp(RNAi) *and untreated N2 animals. However this effect is minor compared to the severe depletion of histone proteins in *cdl-1(RNAi) *animals. This effect of *cdl-1(RNAi) *on histone protein synthesis indicates that *cdl-1*, as is the case for its homologues in other animals, is mainly required for post-transcriptional control of histone gene expression.

### *C. elegans *histone genes are expressed during periods of active cell division

**Figure 5:**
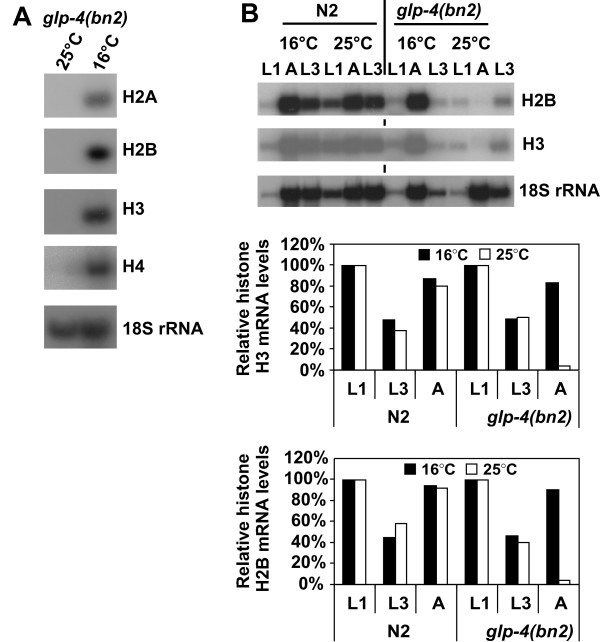
**Histone gene expression is developmentally regulated and occurs mainly during early development and in the germ line**. (A) *glp-4(bn2) *animals were grown at 16°C or 25°C. RNA was isolated from adult animals and histone mRNA levels were analysed by Northern blotting as described. 18S rRNA was detected to control for equal loading. (B) N2 and *glp-4(bn2) *embryos were grown at 16°C or 25°C to L1, L3 and adult (A) stages. RNA was isolated and analysed by Northern blotting, and histone mRNA levels were determined as described. Blots were stripped between probing with histone mRNAs. For the graph, histone mRNA levels were standardised with respect to 18S rRNA, and are shown as percentage of histone mRNA in L1 animals, which was defined as 100 %.

It is well established that replication-dependent histone gene expression occurs in mitotically active cells and is coupled to DNA synthesis. With this in mind we investigated the correlation between histone gene expression and cell division throughout *C. elegans *development. We examined the level of histone transcripts from wild-type (N2) and *glp-4(bn2) *hermaphrodites by Northern Blotting. Hermaphrodites homozygous for the temperature-sensitive *glp-4(bn2) *mutation almost completely lack germ line proliferation when grown at the restrictive temperature of 25°C [32]. Histone H2A, H2B, H3 and H4 transcripts cannot be detected in *glp-4(bn2) *adult hermaphrodites grown at the restrictive temperature (Fig. 5A), but are clearly present in those grown at the permissive temperature. As these animals lack a germ line, this suggests that histone gene expression in *C. elegans *adults is largely confined to the germ line; the only actively dividing cells at this stage of development. If this were the case we would expect to see histone gene expression in earlier, larval stages, when many somatic cell lineages are actively dividing, irrespective of whether they have a germ line. We therefore examined histone gene expression in *glp-4(bn2) *and Bristol (N2) worms at the first and third larval stages and in adults (L1, L3 and A, respectively) at the permissive (16°C) and non-permissive (25°C) temperatures (Fig. 5B). *glp-4(bn2) *worms grown at the permissive temperature did not differ from wild-type in their levels of histone gene expression (Fig. 5B, histograms). Similarly, there was no significant difference in the histone H2B and H3 RNA levels between L1 and L3 *glp-4(bn2) *and wild type larvae grown at the restrictive temperature, consistent with the fact that most of the dividing cells in these developmental stages are somatic cells. In agreement with the results seen in Figure 5A, the level of histone gene expression in *glp-4(bn2) *adult worms raised at the restrictive temperature is negligible (Fig. 5B). The lack of histone gene expression in these animals is particularly evident when compared to the level of 18S rRNA. These results indicate that, consistent with the role of histones in the cell nucleus, histone gene expression in *C. elegans *occurs during periods of active cell division.

## Discussion

The organisation and structure of *C. elegans *histone genes is similar to histone genes in other metazoans. The conserved hairpin element in the 3' UTR, the core element of the binding site for HBP/SLBP, is clearly present, and is followed by a conserved element that may serve as the U7 snRNP binding site HDE (Fig. 1 and additional file 1). However all *C. elegans *histone genes have a conserved potential polyadenylation signal immediately downstream of the HDE, a feature not shared with histone genes from other organisms. We have used three independent approaches to analyse the structure of histone mRNA 3' ends. They reveal that histone mRNAs end after the hairpin structure, indicating that 3' end processing of *C. elegans *histone mRNAs occurs via cleavage immediately after the hairpin, and that cleavage following the putative polyadenylation signal occurs rarely, if at all. First, we found that *C. elegans *histone mRNAs were lost during selection of poly(A) mRNA using oligo-dT cellulose, similar to HeLa cell histone mRNA known to end after the hairpin and to lack a poly(A) tail (Fig. 2). Next, we investigated the structure of histone mRNA ends using an RNase protection assay designed to distinguish between RNAs ending after the hairpin, and RNAs ending after the polyadenylation signal (Fig. 3). The protection patterns obtained indicate that the vast majority of transcripts of the histone genes analysed (*his-9, his-13*, *his-42 *and *his-62*) end after the hairpin signal. Finally, cloning and sequencing of synthetically poly(I/A) tailed histone mRNAs showed that none of the mRNAs included the putative HDE element or polyadenylation signal (Table 1). Instead this revealed that histone mRNAs end three to six nucleotides after the hairpin structure and, as they are a substrate for poly(A) polymerase, have a 3'OH group. Thus our data indicate that the structure of *C. elegans *histone mRNA 3' ends is similar to the structure of histone mRNA 3' ends in vertebrates and *Drosophila *[19,20], and is typical for histone-specific mRNA 3' end processing mediated by the U7snRNP and associated factors. It will be interesting to analyse the function of the conserved AATCC element. We believe that this is the core motif of a U7 snRNP binding site. We will exploit this to search for U7 snRNA, using bioinformatics and experimental approaches.

It is known that polyadenylation signals contribute to transcription termination by changing the properties of the passing RNA polymerase [33]. Perhaps, in *C. elegans *replication-dependent histone genes the polyadenylation signal functions as transcription terminator rather then as an RNA processing site. We also cannot currently exclude the possibility that, instead of histone-specific processing, the mRNA 3' ends are formed by cleavage after the polyadenylation signal, followed by 3'-5' exonucleolytic trimming of the pre-mRNA up to the hairpin element.

We previously described that *cdl-1(RNAi) *results in the inhibition of histone synthesis [13]. Here we analyse this in more detail and show that *cdl-1(RNAi) *does not significantly affect histone mRNA levels, but, as shown previously, severely inhibits histone protein expression (Fig. 4). This indicates that CDL-1 functions similar to HBP/SLBP in mammals and in *Drosophila*. In these animals, HBP/SLBP controls post-transcriptional regulation of histone gene expression including mRNA 3' end processing and translation. It will be interesting to determine whether similar to the role of HBP/SLBP in other organisms, CDL-1 is required for histone mRNA 3' end processing, translation and perhaps also mRNA stability control in *C. elegans*.

We have shown that during *C. elegans *development, histone mRNA levels positively correlate with mitotically active tissues (Fig. 5). Thus, as in other organisms, *C. elegans *histone gene expression is cell cycle regulated. In wild-type embryos we detected a two-fold reduction of histone mRNA levels, relative to 18S rRNA, in L3-stage animals compared to L1 larvae, presumably reflecting the fact that proportionally more cells are proliferating in L1 larvae compared to L3 larvae. Relative histone mRNA levels were again increased in adult N2 animals, which was expected since the adult germ line is mitotically active, and most adults would contain a number of highly proliferating early embryos. This pattern was independent of temperature and was similar at 16°C and 25°C. The fact that no histone mRNAs could be detected from *glp4(bn2) *adult animals grown at 25 °C, which essentially lack germ line proliferation when grown at the restrictive temperature, shows that in adults most, if not all histone gene expression is confined to the germ line. Thus our findings indicate that the link between DNA synthesis and histone gene expression observed in mammals and *Drosophila *is also the case for *C. elegans*.

## Conclusion

*C. elegans *is a well established animal model to study development. In contrast, comparatively little is known about the control of gene expression in this animal. Here we show that histone gene expression is linked to cell proliferation, and is subject to similar post-transcriptional controls as histone gene expression in other animal species. Thus *C. elegans *will be an ideal model to investigate the regulation of histone gene expression during animal development.

## Methods

### Bioinformatics

Histone gene 3'UTR sequences were downloaded from the Wormbase database [34] and aligned using ClustalW [35].

### Nematode strains and culturing

Standard *C. elegans *culturing techniques were used [36]. N2 (Bristol) was used as wild type. The *glp-4 *temperature sensitive (ts) mutant [32], which is defective in germ line proliferation at the restrictive temperature of 25°C, was generously supplied by the *Caenorhabditis *Genetics Center. Strains were grown at 16°C and 25°C as indicated in the text.

### RNAi treatments

RNAi by feeding was carried out as described previously [13]. The *cdl-1(RNAi) *construct was described earlier [13]. GFP RNAi was performed using plasmid pPD128.110 (Fire Vector kit 1999 [37], Addgene plasmid 1649).

### RT-PCR

To confirm gene knockdown by RT-PCR, 1 μg of total RNA was reverse transcribed using random primers (Retroscript kit, Ambion) and subsequent PCR was performed using Taq DNA polymerase (Promega). *cdl-1 *and *act-1 *RT-PCR was performed using primers CGTAAAGCACCACGAGGCCGTC and GACGACATCTTGGAGAAGTCAGTTG, and CGTGGTTACTCTTTCACCACCACCGCT and GGACTCGTCGTATTCTTGCTTGGAGAT respectively. 21, 22 and 23 PCR cycles were performed using an annealing temperature of 55°C, and extension time of 1 minute for all reactions. Products were analysed by agarose gel electrophoresis, visualised by staining with ethidium bromide and quantitated using a Molecular Imager Gel Doc XR System (Biorad) and analysed using AIDA Image Analyzer Software (Raytest). To determine the efficiency of *cdl-1 *knockdown we standardised the *cdl-1*signal with respect to the corresponding *actin *signal. The average of the measurements with N2 RNA was defined as 100%. The standard deviations were calculated from the measurements made for RNA from N2, *gfp(RNAi) *and *cdl-1(RNAi) *animals, respectively.

### Developmental time course

N2 and *glp-4(bn2) *embryos were obtained from bleaching gravid adult hermaphrodites, aliquoted onto NGM-OP50 plates and incubated at 16°C or 25°C. Worms were harvested from plates by gentle washing with sterile M9 salts. L1 larvae were harvested after 15 hours and 30 hours at 25°C and 16°C, respectively; L3 larvae were harvested following 36 hours and 72 hours at 25°C and 16°C, respectively; and adult animals were harvested after 72 hours and 144 hours at 25°C and 16°C, respectively.

### RNA extraction

Pelleted worms were snap-frozen in 250 μl Trizol reagent (Invitrogen) in liquid nitrogen, thawed at 42°C and vortexed for 30 seconds. Following five cycles of freeze-thawing, 50 μl chloroform was added and mixture incubated at room temperature for 3 minutes, spun at 4°C for 20 minutes at 13200 rpm in a table top centrifuge and clear phase containing RNA removed. Total RNA was isopropanol precipitated and stored in 75% ethanol (nuclease free) until required. RNA concentration was determined using a 1 cm cuvette assuming 1 OD_260 nm _corresponds to 40 μg RNA.

### Selection of poly(A) RNA

200 μg total RNA from *C.elegans *or HeLa cells was subjected to poly(A) selection with oligo-dT cellulose (Poly(A)Purist, Ambion) as per manufacturer's instructions. Half of the poly(A) enriched fraction was subjected to a second round of selection. Poly(A) selected RNA was precipitated with ethanol and resuspended in 10 μl DEPC treated water.

### Northern blotting

RNA was fractionated on 1.2 % or 1.5 % agarose gels and transferred to Hybond N membranes as described [10]. Probes to detect human histone genes were described earlier [10]. Blots were stripped between probings as recommended by the manufacturer. The human GAPDH probe spans nucleotides 2041–3239 of the open reading and was PCR amplified from genomic DNA, inserted into pGEM-T easy and subsequently released by restriction digestion using *Eco*RI. The *C. elegans *histone H4 probe was prepared by PCR using a cloned *his-10 *gene as template and spans nucleotides 9 – 312 of the *his-10 *ORF. The *C. elegans *histone H3 probe was prepared by PCR using a cloned *his-9 *gene as template and spans nucleotides 1 – 398 of the *his-9 *ORF. Probes detecting histone H2A (*his-30*) and H2B (*his-62*) and actin (*act-1*) mRNA were prepared by RT-PCR from Bristol (N2) RNA and span nucleotides 1 – 372, 9 – 366 and 589–1096 of the respective open reading frames. The probe detecting *C. elegans *GAPDH (*gdp-1*) mRNA was amplified from genomic DNA and spans nucleotides 19–995 of the *gpd-1 *open reading frame. This probe has high sequence identity to GAPDH *gpd-2*,*gpd-3 *and *gpd-4 *genes and cannot distinguish between transcripts from these genes. Probes were labelled with ^32^P-dCTP using a random prime labelling kit (Roche). *C. elegans *18S rRNA was detected using the oligonucleotide ACGTATCTAATCGCCTTCGTTCC ^32^P end labelled using T4 polynucleotide kinase and γ-^32^PATP. Data was analysed by autoradiography and using a Fuji Phosphoimager and AIDA Image Analyzer Software (Raytest).

### RNase protection assays

The probe to detect the *his-62 *gene spans nucleotides 3 – 145 of the *his-62 *3' UTR. The template for transcription was amplified from genomic DNA using primers CATTGGCTGAAAACTAACC and TAATACGACTCACTATAGGG*GAATCCAACG*TTTCAAGAAAAGAC. This produced a template for T7 RNA polymerase transcription containing the T7 RNA polymerase promoter followed by 10 non-histone specific bases prior to sequence complementary to *his-62 *mRNA. The probe to detect *his-9 *mRNA spans from nucleotide 400 in the *his-9 *ORF to nucleotide 205 of the 3' UTR. The template for transcription was amplified from genomic DNA using the primers AGCGTGCTTAAATGCCTTCC and TAATACGACTCACTATAGGGCGAATCGTACGCTGATAGCTTATGTTCAAAAACTGAGTA, resulting in a product containing T7 RNA polymerase promoter followed by 20 non-histone specific nucleotides and 216 nucleotides *his-9 *sequence. The PCR product was inserted into pGEM-T easy and sequenced (DNA Sequencing Service, Dundee University). For RNA synthesis, the fragment was re-amplified using the same primers and transcribed using the T7 RNA polymerase to produce a probe complementary to *his-9 *mRNA. Uniformly labelled RNA was prepared by *in vitro *transcription using T7 RNA polymerase and α^32^P-UTP. RNase protection assays were normally done with 12.5 μg RNA using RNases A and T1 (RPA II kit, Ambion). ^32^P-labelled pBR322 DNA/*Msp*I marker was prepared by endlabelling using a ^32^P-dCTP and Klenow fragment. 5'^32^P-labelled *his-62 *probe RNA was prepared by treating non-labelled RNA with alkaline phosphatase, followed by end-labelling with T4 polynucleotide kinase and γ-^32^PATP. The KOH ladder was produced using 5' ^32^P end-labelled probe as described [38]. Reactions were analysed by 8% polyacrylamide/7M urea gel electrophoresis and products were visualised by autoradiography.

### Sequencing of histone mRNA 3' ends

RNA tailing with yeast poly(A) polymerase was done essentially as described [28]. 10 μg total RNA from N2 adults was incubated for 20 minutes at 37°C with 0.5 mM ITP and 60 units yeast poly(A) polymerase (GE Healthcare). Then ATP was added to a concentration of 0.5 mM and incubation continued for 20 minutes, and then stopped by 10 minutes incubation at 65°C. RNA was reverse transcribed with M-MLV reverse transcriptase (Promega) using an oligo-dT-anchor primer (GCGAGCTCCGCGGCCGCGTTTTTTTTTTTTTTT), and histone gene UTR fragments were amplified by PCR using gene specific fwd primers (AGCGTGCTTAAATGCCTTCC, TGGTGGAGACAAGGAATAA, CCAAGTACACCTCCAGCAAG, and CTCTGTACGGATTCGGAGGAT for H3, H2A, H2B and H4 histones respectively) in combination with the anchor primer GCGAGCTCCGCGGCCGCG. PCR products were gel purified, cloned into pGEM-T easy (Promega) and sequenced.

## Authors' contributions

RK carried out the sequencing of histone mRNA ends, the RNAi experiments and helped draft the manuscript. SW carried out sequence alignments, poly(A) selection and RNAse mapping of histone mRNA 3' ends. Both SW and RK carried out experiments analysing histone gene expression during development. JP participated in the conception and design of the study and helped draft the manuscript. BM conceived of the study, and participated in its design and coordination and drafted the manuscript. All authors read and approved the final manuscript.

## Supplementary Material

Additional file 1 Alignment of *C. elegans *histone mRNA 3'UTR sequences. Sequence alignments used to identify consensus 3'UTR elements of histone mRNAs shown in Figure 1. Click here for file

Additional file 2 **Effect of *cdl-1*(RNAi) treatment on the 3' end of *his-*62 mRNA**. Internally ^32^P labelled *his-62 *probe was hybridised with either total RNA from adult N2 animals, or total RNA from *cdl-1(RNAi) *adults, or yeast RNA and subjected to treatment with RNase A/T1 as described. Samples were analysed by denaturing PAGE and visualised by autoradiography. The asterisk marks probe protected by hybridisation to template DNA. Note that the protection patterns obtained with N2 and *cdl-1(RNAi) *treatment are identical and that *cdl-1(RNAi) *does not result in longer protected fragments diagnostic for end-formation using the polyadenylation signal. Click here for file
