# Supplementary material for: Histone gene expression and histone mRNA 3' end structure in Caenorhabditis elegans
Source: BMC Mol Biol. 2007 Jun 14;8:51. doi: 10.1186/1471-2199-8-51 (PMC1924863; doi:10.1186/1471-2199-8-51)
Supplement: Additional file 1 — Alignment of C. elegans histone mRNA 3'UTR sequences. Sequence alignments used to identify consensus 3'UTR elements of histone mRNAs shown in Figure 1. [file 1471-2199-8-51-S1.pdf]

```
H2A
his-30     1 ---ATACTTGTCTCCAAATAATACAAACCGAACCCAACGGCCCTCTTTAGGGCCACATTTACAATAAATCCTCTAACATCTTA--TT--AATAAACTTT-TTTTCGAA---------CATTTATT-TTTTC-TGGCTCGGAAAACTCATATATGCACAA----TATAGATTATGCATT-CAATTATCTTCTTTTC-A-CTTTTGTCAGTTTACTACC---
his-33     1 ---ATACTTGTCTCCAAATAATACAAACCGAACCCAACGGCCCTCTTTAGGGCCACATTTACAATAAATCCTCTAACATCTTA--TT--AATAAACTTT-TTTTCGAA---------CATTTATT-TTTTC-TGGCTCGGAAAACTCATATATGCACAA----TATAGATTATGCATT-CAATTATCTTCTTTTC-A-CTTTTGTCAGTTTACTACC---
his-21     1 ---ATGCCT-TCTTAAA-ACAT-TTTACCGAACCCAACGGCCCTCTTTAGGGCCACAAATATTTCAAATCCTCT---ATTGAC--TG--AATAAATATA-TTATAGTATT--T----CAATTTTT-TTTT--TGTACTTTATTACTAATTGAAAATCTT----TTCAACGTTTGCAAT-CAAGAAACTTCTTCTCGA-AATTAGTTGATTCACAAAA---
his-53     1 ---ATGCCT-TCTTAAA-ACAT-TTTACCGAACCCAACGGCCCTCTTTAGAGCCACAAATATTTCAAATCCTCT---ATTGAC--TG--AATAAATATA-TTATAGTATT--T----CAATTTTT-TTTT--TGTACTTTATTACTAATTGAAAATCTT----TTCAACGTTTGCAAT-CAAGAAACTTCTTCTCGA-AATTAGTTGATTCACAAAA---
his-19     1 ---ATGCCT-TCTTAAA-ACAT-TTTACCGAACCCAACGGCCCTCTTTAGAGCCACAAATATTTCAAATCCTCT---ATTGAC--TG--AATAAATATA-TTATAGTATT--T----CAATTTTT-TTTT--TGTACTTTATTACTAATTGAAAATCTT----TTCAACGTTTGCAAT-CAAGAAACTTCTTCTCGA-AATTAGTTGATTCACAAAA---
his-12     1 -----GCAT-CCTTCAACACAC-CTAACCGAACCCAACGGCCCTCTTTAGGGCCACAAATGTTATAAATCCTATT--ATAAAC--TG--AATAAATCT---TATCAAA---------CATGTTTT-GCAT--TG--CCATAATATTACCTGAAAAACTGAAAATTAAGAAATTCGTAT-CTA-AAATTTAGAATAGT-CACCAGTATCATCAGGAAC---
his-43     1 -----GCAT-CCTTCAACACAC-CTAACCGAACCCAACGGCCCTCTTTAGGGCCACAAATGTTATAAATCCTATT--ATAAAC--TG--AATAAATCT---TATCAAA---------CATGTTTT-GCAT--TG--CCATAATATTACCTGAAAAACTGAAAATTAAGAAATTCGTAT-CTA-AAATTTAGAATAGT-CACCAGTATCATCAGGAAC---
his-3      1 ---ATGCCT-TCTTAAA-ACAT-TTTACCGAACCCAACGGCCCTCTTTAGGGCCACAAATATTTAAAATCCTCT---ATTGAACATG--AATAAAAATT-ATTTT-TAT--------CAATGGTT-TTTGCACGTACTTTATTATAAATTGAAAATC-----------GTTTGCAAA-GAAGAAACTTCTTTTTGG-ATTTGGTTGATTCACAAAAC-
his-16     1 ------CAC-TCTTCAACACAC-CTAACCGAACCCAACGGCCCTCTTTAGGGCCACAAATGTTCCAAATCCATTTATATTTGCCTTGTAAATAAAACCA-AAACCGAAAA--TTCATCATTTATTCTTTTCATG--ATTCCGTTGTACTTGAAACACTTGGTCGTCT--CTCTGGCAC-CAC--AATTT-TTATG---CATT--TTCACACAAAAATG--
his-57     1 GAACTTTCT-TCTTTAA-ACAAACCCACCGAACCCAACGGCCCTCTTTAGGGCCACAAATATCCAAAAACCTGACA-ATCCCATTTT--AATAAAGTTACATTTATTA---------CATTTATT--TTGAATAATCTGTTCGCTTGAATTCAATATTG----TT---ATTATGTTTC-CACATAAATT--CAACG---ATCAGTTCGAATACGATATA-
his-65     1 GAACTTTCT-TCTTTAA-ACAAACCCACCGAACCCAACGGCCCTCTTTAGGGCCACAAATATAAAAAATCCTAA-AGATATTTCAT---ATTACATTTA--TTTTGAAT---------AATATGTT-------TG--CTTGAATTCAATCAGTATTACTG----TC---AATAAGTTTT-CAC--ATGTT-TTCGAGC-CAAGATATCGGTCAGGAAAC--
his-47     1 GAACTTTCT-TCTTTAA-ACAAACCCACCGAACCCAACGGCC-TCTTTAGGGCCACAAATATACAAAATCCTACT--TTTTCCCATT--AATAAATTTTTTAATAAAGTTCATATTACATTTATT--TTTAATAAGTTGTTTGCTTGAATGCAATATTGT---TATAAAGTTTTCTAC-CTTCCATGTTCTGTCAGA-AAAC-GT---ATGAGGAAAT--
his-7      1 ---ATGCCT-TCTTAAA-ACAA-CTTACCGAACCCAACGGCCCTCTTTAGGGC-ACAAATATCTTAA-TCCTGA---GTTAAA--TGTAAATAAAATTGTATTTT-AAT---------AATGTTT------ATTAGTTGTTGTATATATTGAAATGATT----TTAAGCAAATGAAGT-GAGAAATTTTGGTACATTGCTCTAGTCTACGTTCAAATGTG
his-61     1 GAACTTTTT-TCTTAAA---AACCCAACCGAACCCAACGGCCCTCTTTAGGGCCACAATTTATCTGAAATCCTAAC-TATCAAT--TG--AATAAATTGTTTTTTATTATT------ATAAATCTT----GAAGACACGTTAATAGTGAATGTTGAACGAAATTGTGATAATTCGAAAAGCTATCAACTTC-AATC-----CTGTTTGATTCACTTTT---
consensus  1     t   t tCTt AA acA     ACCGAACCCAACGGCCcTCTTTAGgGCcACAaaTat   AAatCCt      aT       T   AaTAaA  t   t t   a         cA  t TT       tg           ta  tg ag  c       t       t tg      c    A  TT               gt  g t a aa

H2B:
his-34     1 GCAATCAAATGCTCAATGCT---AATTACA-CCGAACCCAACGGCCCTCTTTAGGGCCACAA-TACATCTAAAATCCATCAG-TTGTTCCAA-TAAAATTTTTAGTAAT----ATTTGTGA-CGTCATGAAAAC-CGT-TAGTTTTAGTTCAATTTTTTTA----AATTTCATTTTAA-ATTT--AT--TATATATGTATCTTAATCA---TGTATTAGC
his-29     1 GCAATCAAATGCTCAATGCT---AATTACA-CCGAACCCAACGGCCCTCTTTAGGGCCACAA-TACATCTAAAATCCATCAG-TTGTTCCAA-TAAAATTTTTAGTAAT----ATTTGTGA-CGTCATGAAAAC-CGT-TAGTTTTAGTTCAATTTTTTTA----AATTTCATTTTAA-ATTT--AT--TATATATGTATCTTAATCA---TGTATTAGC
his-52     1 GCATTCAATTGCTGAAAGCA---AAT--CA-CCGAACCCAACGGCCCTCTTAAGGGCCACAAATGTAT-TAAAATCC------TTTTTCAAT-TAGTAGTTTATTTAATG--AAATAAAGT-TTTCTTCATTCTTCAT-CACTATTATTCTTATTTTTTCT------TCTTATTTAAA-ATCT-GAT--CAAATAATTGCC--AATGAAA-TAAATTTCT
his-22     1 GCATTCAATTGCTGAAAGCA---AAT--CA-CCGAACCCAACGGCCCTCTTAAGGGCCACAAATGTAT-TAAAATCC------TTTTTCAAT-TAGTAGTTTATTTAATG--AAATAAAGT-TTTCTTCATTCTTCAT-CACTATTATTCTTATTTTTTCT------TCTTATTTAAA-ATCT-GAT--CAAATAATTGCC--AATGAAA-TAAATTTCT
his-54     1 GCATTCAATTGCTGAAAGCA---AAT--CA-CCGAACCCAACGGCCCTCTTAAGGGCCACAAATGTAT-TAAAATCC------TTTTTCAAT-TAGTAGTTTATTTAATG--AAATAAAGT-TTTTATAAATAGACA--CAATACAACAGTTGTGCCACAAAC--AATCCTTTTTCAG-ATATCGATGCCAAATATGTGTC--AGTTA---TTATTTTCC
his-20     1 GCATTCAATTGCTGAAAGCA---AAT--CA-CCGAACCCAACGGCCCTCTTAAGGGCCACAAATGTAT-TAAAATCC------TTTTTCAAT-TAGTAGTTTATTTAATG--AAATAAAGT-TTTTATAAATAGACA--CAATACAACAGTTGTGCCACAAAC--AATCCTTTTTCAG-ATATCGATGCCAAATATGTGTC--AGTTA---TTATTTTCC
his-48     1 GCCATTA--GGCTGAAAACT---ACCC-CA-CCGAACCCAACGGCCCTCTTTAGGGCCACAAATCT---TATAATCC------TATGTTATT-AACCAAGTGAATAAAT----AATTTTCA-ATT-ATACTCTTTCA--TAATTTTCAGGTTTTGCATTTTCC--AAGTTCAGTT-AA-TTTGAGAT--TAACCCAGTATT--CATTTTC-TAAAAGTCA
his-58     1 GCCATTA--GGCTGAAAACT---ACCC-CA-CCGAACCCAACGGCCCTCTTTAGGGCCACAAATCT---TATAATCC------TATGTTATT-AACCAAGTGAATGAAT----AATTTTCA-ATT-ATACTCTTTCA--TAATTTTCAGGTTTTGCATTTTCC--AAGTTCAGTT-AA-TTTGAGAT--TAACCCAGTATT--CATTTTC-TAAAAGTCA
his-39     1 GCATTCAATTGCTGAAAGCA---AAT--CA-CCGAACCCAACGGCCCTCTTTAGAGCCACAAATGTA-CTAAAATCC------ATTATCAATATAAATATTTGTACAGTTTAAATTCTTGT-GTTTATATATTTGGCTTTCAATTGTATTCAGATGCAATAT------GAATAATT----CTTTGAAT---AATTCATTATC--AATCACATTTTCTTTAC
his-8      1 GCATTCAATTGCTGAAAGCA---AAT--CA-CCGAACCCAACGGCCCTCTTTAGGGCCACATTTGT---GAAAATCC------TTTATCAATGCAAATATTCGTATAATTTCGATTATATT-CATCAAACTGGT--ATTCAGTTTTGTATTGTGGCGATTC-----ATCAAACAC-AC-ATTCAGAAAATAAACATTTTT---ATGTACTTGTTCTATCA
his-66     1 GCCATTA--GGCTGAAAACT---ACCC-CA-CCGAACCCAACGGCCCTCTTTAGGGCCACAAATCTT--TAAAATCCTATAGACTTGATAATCAATAAATCGTTTTAATTTTGACTAAA---TCTCATTTTGTTT-ATGGTGTCTTGTTATT-TGCTGTCATG--AGGCTTTTTCTTA-CTGG--ACCGCACAGC-GCCTCA-AATCGGGAGAGTTAAGA
his-62     1 GCCATTG---GCTGAAAACT---AACC-AA-CCGAACCCAACGGCCCTCTTTAGGGCCACAAATAT---TGAAATCC-------TTACAAACTACATGTTCGT-TAATA--AATTATATGCACTCTTATATATTCAC-GTCTTTTATATTAGGTCTTTTCTTGAAATCTTAATCTAGTATTACGTT--TTCCTAGCCTTCGAAGGAATTCAAGAGTTCG
his-44     1 GCCATTC--GGCTGAAAATGTTGAACAACAACCGAACCCAACGGCCCTCTTTAGGGCCACACATGA--CAAAAATCC--------GAATATTCTTCTTGTTGAT-AAAT----ATTATT----TGCATCGGGAGT--TTCTGTTTTAATAATTAGATTTTACAGACATTATTTATTTCATTATGCATACAAACGCTACAGCAAACAGCAATTTAAAAACA
his-4      1 -CATTCAATTGCTGAAAGC----AATCTAA-CCGAACCCAACGGCCCTCTTTAGGGCCACATTTTTA---AAAATCCA-----ATTTTTCTT---CT-GTTTTTAAACTG--AAATAAAGT-TTTTACGAATATGAGGATAGTTTTTAAAATTAATTTTTA-----ATTTAATTT-----TTAATATT-TTTTGTTTTTAATAATGTTTGTTTATTTTT
consensus  1 gC  T a   GCTgAAagc    Aa   cA CCGAACCCAACGGCCCTCTT AGgGCCACAa T t   taaAATCC        tt t a t  a   gtt     AaT   A T        t at        g   agT tta            t       a      tt      T    at   a       t t   aat      t

H3
his-27     1 ATTTCTGATTTTCAGA--GTTTGAGACCGTTTCGATTCAAACCCCCACCGAACCCAACGGCCCTCTTTAGGGCCACATAT---CTATGATCCATCGAATTTACTTTGAATAAA-AATTGCC---ATCTTTTCTTGTATAGT-TCTAAAATTTTTCTTA---GCAGC-TGTTAAGAAAAC-----AAAACATTTT-----TACATTTTACTAAAACAT-TT
his-49     1 ATTTCTGATTTTCAGA--GTTTGAGACCGTTTCGATTCAAACCCCCACCGAACCCAACGGCCCTCTTTAGGGCCACATAT---CTATGATCCATCGAATTTACTTTGAATAAA-AATTGCC---ATCTTTTCTTGTATAGT-TCTAAAATTTTTCTTA---GCAGC-TGTTAAGAAAAC-----AAAACATTTT-----TACATTTTACTAAAACAT-TT
his-17     1 ATTTCTGATTTTCAGA--GTTTGAGACCGTTTCGATTCAAACCCCCACCGAACCCAACGGCCCTCTTTAGGGCCACATAT---CTATGATCCATCGAATTTACTTTGAATAAA-AATTGCC---ATCTTTTCTTGTATAGT-TCTAAAATTTTTCTTA---GCAGC-TGTTAAGAAAAC-----AAAACATTTT-----TACATTTTACTAAAACAT-TT
his-2      1 ATTTCTGATTTTCAGAAAGTTTGAGACCGTTTCAATTCAAACCCC-ACCGAACCCAACGGCCCTCTTTAGGGCCACATAC---ATATGATCCA-----ATCATTTTCAATCCA-ATTTGA----ATAAATTATTCATTATT-TTTTAACTTATTAAAA---AAAAA-GAACAAAACCAG-----GAAACTTTAGAGCCATACAATTTAGTATATTTT-TA
his-6      1 ATTTCTGATTTCCAGA--GTTTGAGACCGTTTCGATTCAAACCCCCACCGAACCCAACGGCCCTCTTTAGGGCCACATAT---CTATTATCCG-----ATCATGTTCAATCCA-ATCTGAATAAATATTATCTTGCGTCGT-T-TTAAATTGTTCGTAT--ACTTC-TGTCGATCTGCCTGTCAAAACATCTCA--AATAAATGTCAAAGATCCAT-T-
his-45     1 ATACCT--TTTTTGGT--AGTTGAGCAAGCTTAATCTCAAAACTTAACCGAACCCAACGGCCCTCTTTAGGGCCACAAATATCCTCAAATCCAT---TTTAATATTCAATGAATACTTTTTTCAATTTCAATTTTTTTAAA-TTTTAAATCTTTCGGAC--ATAGT-GGTTCCGTGTAT-----A-----TTATA---ATATGATTGATTCAAAGAT-TT
his-9      1 ATGCCT----TCCAATGGTACTGAGCCAGCTTGATCTCAAAATCTAACCGAACCCAACGGCCCTCTTTAGGGCCACAAATGTAATATAATCCGGC---TTGATTTCTAATAAA--TTTGTATTGTTGTTTTTGTCATTGTGTATTGAATATTTCTCGA--AATAT-TGTGATGAATAA----AA----TTATAT---TACTCAGTTTTGAACATATG
his-42     1 ATGCCT----TCCAATGGTACTGAGCCAGCTTGATCTCAAAATCTAACCGAACCCAACGGCCCTCTTTAGGGCCACAAATGTAATATAATCCGGC---TTGATTTCTAATAAA--TTTGTATTGTTGTTTTTGTCATTGTGTATTGAATATTTCTAGTGTACACT-TTTGAAATTTAG-----T-----TCATT----TA---TTTAATGGCAAAAGTA
his-13     1 ATGCCT----TCCAATGGTACTGAGCCAGCTTGATCTCAAAATCTAACCGAACCCAACGGCCCTCTTTAGGGCCACAAATGTAATATAATCCGGC---TTGATTTCTAATAAA--TTTGTATTGTTGTTTTTGTCATTGTGTATTGAATATTTCTAGTGTACACT-TTTGAAATTTAG-----T-----TCATT----TA---TTTAATGGCAAAAGTA
his-55     1 ATACCT--TTTTTGGT--AGTTGAGCAAGCTTAATCTCAAAACTTAACCGAACCCAACGGCCCTCTTTAGGGCCACAAATG-CATA-AATCCAGTC--CTTGTGTAAAATAAA----------ATTGTTTCTTTATTTGTTTTTGTATCATTTGAA--GGCCC-GGAGAACCGAACTA-CAAATTCGTCAGAAGTTCGCAGATTTATAAAAAAGGTT
his-63     1 ATACCT--TTTTTGGT--AGTTGAGCAAGCTTAATCTCAAAACTTAACCGAACCCAACGGCCCTCTTTAGGGCCACAAAT-TCATA-AGTCCAG--ATCTTGTCTAAAATAAA--ATCGT--TCTTCTAAGTTTCACTTTT-TTTTAAAGGTTTTACATTTCGCAG-GCTCGAGAAATCGG---G-----TCATT----TG---ATTCTTCAAAAGTCTG
his-59     1 ATACCT----TATGGT--AGTTAAGTAAGCTTCAACTCAAAACTC-ACCGAACCCAACGGCCCTCTTTAGGGCCACAATTTACTGAAAATCCAT---TTTTATGTTTAATAAA----------TACTTTTTTAAATTTGAATTTGACACTATTAGTTT--ACTGT-TATGATTTTTGATTGCTGAACTTTTTTGTTGTAAAAACTTCATTGATAAGTTA
his-32     1 ATGTCT--T-TGTGGATATTTTGTACAAGCTTGATCTCAAA--TA-ACCGAACCCAACGGCCCTCTTTAGGGCCACAACTATCCTT-GAACCTT-GAACAAATTTGAAATAAACTTTT-----ATTGTATTCGTGAACTCA-AATAAATAATTTCTTGTCTATTGTATGTTCTTTGGAATTGGAAC----TTGCAACTTGAAAGTTCAAATTAAAG-T-
consensus  1 AT   CT     T  ag      tTgag   G TT  g  TCAAA       ACCGAACCCAACGGCCCTCTTTAGGGCCACA at     ta aatCCa        t at  T   AATaaA    tt         t t tt t    t gt t  T aaat   TT   a    g      gt a     a      a      T          ta     tt  t a a a  T

H4
his-56     1 ---TTATTATCTTTTCTGTTTTCATACAAACCAAATACCGAACCCAACGGCCCTCTTTAGGGCCACAAATATCCAA--AATCCTAATTG--A-TCTC--GTTAAATAAATCTTAAATA---AT--TTTGATTTTTCTATTTTAGCA-T----TTTCAAATAC-AAGTTGACATGATATTT---TCTTTATT-GCTTTGC---ACAACTACATA-----CT
his-46     1 ---TTATTATCTTTTCTGTTTTCATACAAACCAAATACCGAACCCAACGGCCCTCTTTAGGGCCACAAATATCCAA--AATCCTAATTG--A-TCTC--GTTAAATAAATCTTAAATA---AT--TTTGATTTTTCTATTTTAGCA-T----TTTCAAATAC-AAGTTGACATGATATTT---TCTTTATT-GCTTTGC---ACAACTACATA-----CT
his-64     1 -TTTTATTATCTTTCCTGTTTTCATACAAACCAAATACCGAACCCAACGGCCCTCTTTAGGGCCACAAATATCCAA--AATCCCAATTA--A-TCTC--GTAAAATAAATCTTAATTA---AT--TTTTATCCTTCAATCTCTGCA-T----TCCTACTTTCTAAGAATCTGTCACAATT---GGTTACTT-TCCCCACC--AGAGTTACAT------CT
his-60     1 TTTCTTTTGTCTTTTCTGTTTTCATACAAACCAATTACCGAACCCAACGGCCCTCTTTAGGGCCACAAATATCCAA--AATCCTAATTT--T-TCTT--TGCTAATAAA--------A---AT--GTTT-TGCCACGCTGTATACA-A----TATCA--TAA-ATTTTTTTAAGGTAATT---TCATCATCAGGTTCGATA-AAAATTTAATT-----AA
his-18     1 ----TTTTATCTCTC--GATTTCATACAACAAACCAACCGAACCCAACGGCCCTCTTTAGGGCCACAATT--CAAA--AATCCCAATTT--A-TGT---GTATAATAAATATTA---A---TT--CTTCCTTATTCACTGT-TGCAGA----AACCAAAAAGTACCAATAATAGACAAA-----CATCAACAGGGCTTCT--AGTTTTACACTCATGTCA
his-28     1 ----TTTTATCTCTC--GATTTCATACAACAAACCAACCGAACCCAACGGCCCTCTTTAGGGCCACAATT--CAAA--AATCCCAATTT--A-TGT---GTATAATAAATATTA---A---TTC-TTTGACTTGTGTCTTTTTCCTTTGGTGATCCATATGCTATTATTTGTTGCAGCAT---TATTCTTCTGATATTGGA-TTATTTTCGCT-----CT
his-50     1 ----TTTTATCTCTC--GATTTCATACAACAAACCAACCGAACCCAACGGCCCTCTTTAGGGCCACAATT--CAAA--AATCCCAATTT--A-TGT---GTATAATAAATATTA---A---TTC-TTTGACTTGTGTCTTTTTCCTTTGGTGATCCATATGCTATTATTTGTTGCAGCAT---TATTCTTCTGATATTGGA-TTATTTTCGCT-----CT
his-5      1 ----TTTTATCTCTC--GATTTCATACAACAAACCAACCGAACCCAACGGCCCTCTTTAGGGCCACAAATG-CAATGTAATCCAGTTTT--A-TTT---AATGAATATTTTTTAC-TA---TTC-TTTGCCATTTTATTTTCAGGAGA---ATCTCAGAACAAAATATTATTTTCAAAA----GGTTTTTT-GTCACAGAA-TTAGTAAGATC-----AT
his-1      1 ----TTTTATCTCTC--GATTTCATACAACCAACCAACCGAACCCAACGGCCCTCTTTAGGGCCACAAAT--CTAA--AATCCTAATTT--TCTGTG--CATAAATATGTGTTCCTTACTTATC-CTTG-AGTTTATATTTTTGCCTT--TTTTGCGTTGTTAAAGCAATGTTGAAAAGT---GTCTGCTTCCATACCAGA-ACAGAT-CATAAAGGAGG
his-38     1 ----TTTTATCTCTC--GATTTCATACAAC----CAACCGAACCCAACGGCCCTCTTTAGGGCCACATAT--CTAA--AATCCTATTTT--TCTTTG--CATCATAAAATGTT---TATTTCTT-ATTCACTGTTGCAAATCAGCAAAA--GTTCAATATAAAAACTTTTCTTTGAGTTTGTCTTGTCTTTTTATCCTGTT-GAAACAATACTG-GAAAG
his-10     1 -----GTCCTCTTGCTTCCATGCAATCTACCAAACTACCGAACCCAACGGCCCTCTTTAGGGCCACAAATATTCTG--AATCCAATTATGAATTGTTC-TTTTAATAAATG----------TC-TTTCTTTTTACTTATTTTGTT-T----TAAAATCTTTAACATTTTAAAGCTGGAGGTCTGCAGTACAGACTCTGTACTCAGATTCAT------A-
his-14     1 -----GTCCTCTTGCTTCCATGCAATCTACCAAACTACCGAACCCAACGGCCCTCTTTAGGGCCACAAATATTCTG--AATCCAATTATGAATTGTTC-TTTTAATAAATG----------TC-TTTCTTTTTACTTATTTTGTT-T----TAAAATCTTTAACATTTTAAAGCTGGAGGTCTGCAGTACAGACTCTGTACTCAGATTCATA-------
his-26     1 -----GTCCTCTTGCTTCCATGCAATCTACCAAACTACCGAACCCAACGGCCCTCTTTAGGGCCACAAATATTCTG--AATCCAATTATGAATTGTTC-TTTTAATAAATG----------TC-TTTCTTTTTACTTATTTTGTT-T----TAAAATCTTTAACATTTTAAAGCTGGAGGTCTGCAGTACAGACTCTGTACTCAGATTCATA-------
his-31     1 -TTCATTTATCTTCT--GTTTTTCTGCAAAC---CCACCGAACCCAACGGCCCTCTTTAGGGCCACAAATGATATGA-ATTCTTAATTTGTTTTTTTTATTTTAATAAAATGTGAAAA--TATCAATTCCATGATTTATATACTCGTT---AATATATATTAAACTTATGGAACATAATT--CAAATCAACACTGACTCC--ACATGGTCCTTTGG----
consensus  1        TtaTCT      g tTtcataCaA    a    ACCGAACCCAACGGCCCTCTTTAGGGCCACAaaT      a  AaTCc a Ttt      T T         AatAaat         a     T    TT                T  g              a  t   A     t         a           t                     a  t ca
```

Additional File 1. Alignment of *C. elegans* histone 3'UTR sequences

The first 200 nucleotides of the 3'UTRs of histone H2A, H2B, H3 and H4 genes were retrieved from Wormbase and aligned using ClustalW. Sequences ≥80 % conserved are boxed in black; positions with ≥80 % similar bases (either purines or pyrimidines, respectively), are boxed in gray. In the consensus sequence the hairpin element, the AATCC element and the polyadenylation signal are underlined. Note that in H2B genes the position of the polyadenylation signal varies and is not shown in the consensus. Instead the signals are bold/underlined in the alignment. Similarly, the few polyadenylation signals not fitting the consensus sequence are in shown bold/underlined in the alignments of H2A, H3 and H4 3'UTRS. In animals, cleavage normally occurs 10 - 30 nucleotides 3' of the AAUAAA element, after the sequence CA, and is also dependent on a G/U downstream element 3' of the cleavage site. The position of a CA dinucleotide is ≥80 % conserved in H2A genes and is shown in consensus sequence. In some H2A genes, and in H2B, H3 and H4 genes the position of CA is less well conserved and CA dinculeotides are underlined in the alignments. This is normally followed by a G/T rich region that may encode the G/U downstream element.
