# Supplementary figures and images for: Histone gene expression and histone mRNA 3' end structure in Caenorhabditis elegans
Source: BMC Mol Biol. 2007 Jun 14;8:51. doi: 10.1186/1471-2199-8-51 (PMC1924863; doi:10.1186/1471-2199-8-51)

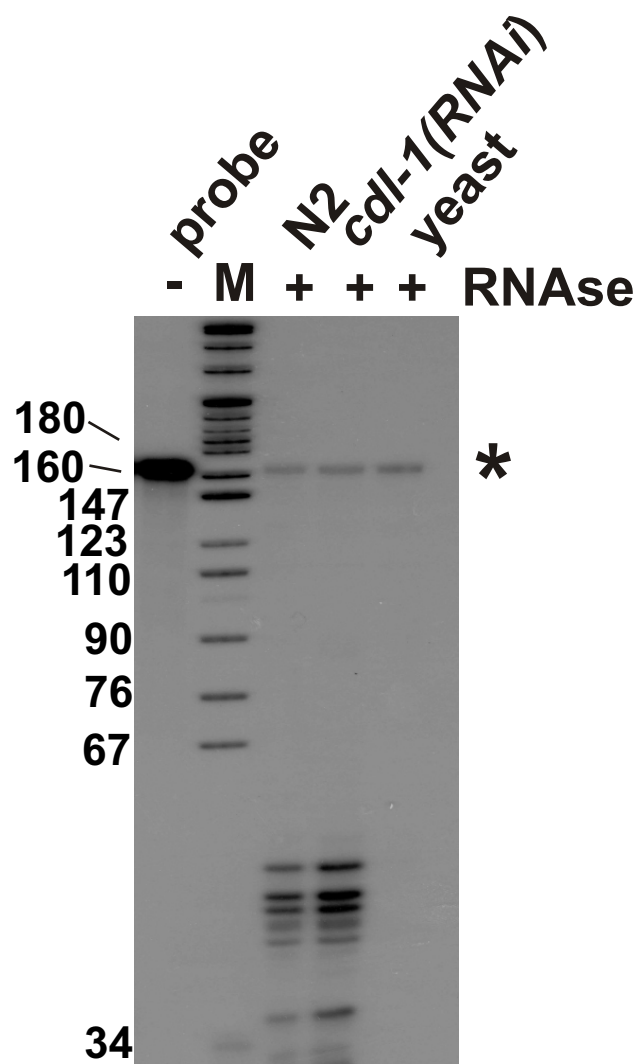

Supplement: Additional file 2 — Effect of cdl-1(RNAi) treatment on the 3' end of his-62 mRNA. Internally 32P labelled his-62 probe was hybridised with either total RNA from adult N2 animals, or total RNA from cdl-1(RNAi) adults, or yeast RNA and subjected to treatment with RNase A/T1 as described. Samples were analysed by denaturing PAGE and visualised by autoradiography. The asterisk marks probe protected by hybridisation to template DNA. Note that the protection patterns obtained with N2 and cdl-1(RNAi) treatment are identical and that cdl-1(RNAi) does not result in longer protected fragments diagnostic for end-formation using the polyadenylation signal. [file 1471-2199-8-51-S2.pdf]
